# Association of objectively measured physical activity with body components in European adolescents

**DOI:** 10.1186/1471-2458-13-667

**Published:** 2013-07-18

**Authors:** David Jiménez-Pavón, Amaya Fernández-Vázquez, Ute Alexy, Raquel Pedrero, Magdalena Cuenca-García, Angela Polito, Jérémy Vanhelst, Yannis Manios, Anthony Kafatos, Dénes Molnar, Michael Sjöström, Luis A Moreno

**Affiliations:** 1 GENUD (Growth, Exercise, Nutrition and Development), Department of Physiotherapy and Nursing, Research Group, Faculty of Health Sciences, University of Zaragoza, Avd. Domingo Miral s/n, CP: 50009, Zaragoza, Spain; 2 Department of Medical Physiology, School of Medicine, University of Granada, Granada, Spain; 3 Rheinische Friedrich-Wilhelms Universität Bonn IEL, Dortmund, Germany; 4 ImFINE Research Group. Faculty of Physical Activity and Sport Sciences-INEF, Technical University of Madrid, Madrid, Spain; 5 Human Nutrition Unit, National Research Institute for Food and Nutrition, Rome, Italy; 6 Inserm U995, University Lille Nord de France, Lille, France; 7 CIC-PT-9301-Inserm-CH&U, University Hospital, Lille, France; 8 Department of Nutrition and Dietetics, Harokopio University, Athens, Greece; 9 Preventive Medicine and Nutrition Clinic, University of Crete, Heraklion, Greece; 10 Department of Paediatrics, University of Pécs, Pécs, Hungary; 11 Unit for preventive nutrition, Department of Biosciences and Nutrition, Karolinska Institutet, Huddinge, Sweden

**Keywords:** Physical activity, Fat mass, Muscular components, Adolescence

## Abstract

**Background:**

Physical activity (PA) is suggested to contribute to fat loss not only through increasing energy expenditure “per se” but also increasing muscle mass; therefore, it would be interesting to better understand the specific associations of PA with the different body’s components such as fat mass and muscle mass. The aim of the present study was to examine the association between objectively measured PA and indices of fat mass and muscle components independently of each other giving, at the same time, gender-specific information in a wide cohort of European adolescents.

**Methods:**

A cross-sectional study in a school setting was conducted in 2200 (1016 males) adolescents (14.7 ±1.2 years). Weight, height, skinfold thickness, bioimpedance and PA (accelerometry) were measured. Indices of fat mass (body mass index, % fat mass, sum of skinfolds) and muscular component (assessed as fat-free mass) were calculated. Multiple regression analyses were performed adjusting for several confounders including fat-free mass and fat mass when possible.

**Results:**

Vigorous PA was positively associated with height (p < 0.05) in males, whilst, vigorous PA, moderate-vigorous PA and average PA were negatively associated with all the indices of fat mass (all p < 0.01) in both genders, except for average PA in relation with body mass index in females. Regarding muscular components, vigorous PA showed positive associations with fat-free mass and muscle mass (all p < 0.05) in both genders. Average PA was positively associated with fat-free mass (both p < 0.05) in males and females.

**Conclusion:**

The present study suggests that PA, especially vigorous PA, is negatively associated with indices of fat mass and positively associated with markers of muscle mass, after adjusting for several confounders (including indices of fat mass and muscle mass when possible). Future studies should focus not only on the classical relationship between PA and fat mass, but also on PA and muscular components, analyzing the independent role of both with the different PA intensities.

## Background

The current global obesity among adolescents [1,2] as well as the beneficial role of physical activity (PA) on fat mass are well known [3]. Moreover, higher levels of moderate to vigorous PA have been found negatively associated with several cardiovascular risk factors including central fat mass [4]. Thus, PA has recently been considered as the main therapeutic tool for metabolic syndrome in children [5]. However, it has been suggested that PA contributes to fat loss not only through increasing energy expenditure “per se” but also by increasing muscle mass, which has a direct effect on metabolic function [5]. Therefore, it would be interesting to better understand the specific associations of PA with the different body’s components such as fat mass and muscle mass.

The relationships between objectively measured PA with indices of fat mass have been analyzed in several reviews [6-10]. In addition, Chung et al. observed that weight status was negatively related to PA in adolescent males and females [11]. It is well known that body mass index (BMI) is an indicator of fat mass but also of lean mass, thus, Metcalf et al. studied the role of height in the associations of BMI and fat mass index (FMI) with several health markers in children aged 7 to 12 years [12]. They concluded that indices of fat mass should take into account height as an important aspect when looking for relationships with health status [12]. In addition, moderate to vigorous PA was negatively associated with adiposity indices after controlling for age, gender, sleep duration, energy intake, sexual maturation, parental socioeconomic status and parental BMI in children aged 8 to 10 years [13]. Finally, others observed that PA fluctuations appear to affect BMI during adolescence (negatively and positively for males and females, respectively) when controlling for baseline fat mass, average number of PA sessions per week, diet and sociodemographic variables [14]. However, none of the previous studies took into account the possible role of fat-free mass (FFM) as a marker of muscle mass in the association between PA and markers of fat mass.

Muscle components have been shown to be associated with several physical fitness components, such as muscle strength in adolescents [15-17]. However, little is known about the specific role of PA levels on FFM [17]. Moliner-Urdiales et al., in a subsample of the Healthy Lifestyle in Europe by Nutrition in Adolescence Cross-Sectional Study (HELENA-CSS), studied the relationship between PA and FFM (using dual-energy X-ray absorptiometry) after controlling for age and pubertal status in both males and females, but they did not find any significant association [17]. Moreover, specific exercise programs have been suggested to promote the maintenance of muscle mass, as an active metabolic tissue, when the aim was weight loss [18,19]. Nevertheless, no studies have been conducted to analyze the association between objectively measured PA and the muscle component independently of several confounders including indices of fat mass in adolescents.

Additionally, socioeconomic status has been associated with obesity and several lifestyle factors [20,21]. Thus, controlling for important confounders such as socioeconomic status is necessary when assessing the relationship between PA, fat mass and muscle mass independently. Moreover, despite the existing literature analyzing the association of PA and markers of fat mass, there are no studies showing the relationships of PA with indices of fat mass and muscle simultaneously as well as with gender-specific information.

The aim of the present study was to examine the association between objectively measured PA and indices of fat mass and FFM (as a marker of muscle mass) independently of each other giving, at the same time, gender-specific information in a wide cohort of European adolescents participating in the HELENA-CSS.

## Methods

The HELENA-CSS is a multi-centre, cross-sectional study performed in ten European cities from 9 countries: Heraklion and Athens (Greece), Dortmund (Germany), Ghent (Belgium), Lille (France), Pécs (Hungary), Rome (Italy), Stockholm (Sweden), Vienna (Austria) and Zaragoza (Spain). This study was designed to obtain reliable and comparable data in a sample of European adolescents. The total sample of the HELENA-CSS was 3546 adolescents with a subset of 2200 (1016 males) adolescents (12.5-17.5 years) with valid data for accelerometers and makers of body composition. Data collection took place from 2006 to 2008 at school setting. Detailed descriptions of the HELENA-CSS sampling and recruitment approaches, standardization and harmonization processes, data collection, analysis strategies, quality control activities, and inclusion criteria have been published elsewhere with a complete description of ethical issues and good clinical practice [22,23].

### Ethics statement

The study protocol was approved by the Ethics Committee at each study centre following the ethical guidelines of the Declaration of Helsinki 1964, the Good Clinical Practice, and the legislation about clinical research in humans. The original names of the ten ethics committees/institutional review boards were 1) Ethics Committee of the Harokopio University from Athens; 2) Ethics Committee of the Medicine’s University from Dortmund; 3) Ethics Committee from Ghent University Hospital; 4) Ethics Committee of the University of Crete School of Medicine from Heraklion; 5) Protection committees people from Lille; 6) A Pecsi Orvostudomanyi és Egészségtudomanyi Központ Regionalis Kutatas-Etikai Bizottsaga from Pècs; 7) Ethics Committee of Medical Activities of the University of Naples Federico II, Naples; 8) Regional Ethics Committee from Stockholm; 9) Ethics Committee of the Medicine’s University from Vienne and 10) Ethics Committee of clinic research of Aragón From Zaragoza. Written informed consent was obtained from the parents (or guardian) and adolescents participating in the study.

### Body composition

The physical examination methods followed in the HELENA-CSS has been described in detail by Nagy et al. [24]. In brief, body height was measured to the nearest 0.1 cm with a stadiometer (SECA 225; SECA, Hamburg, Germany) while adolescents were standing barefoot. Body mass was determined to the nearest 0.05 kg using a balance scale (SECA 861; SECA, Hamburg, Germany) with the subject in their underwear. BMI was calculated as body mass (kg) divided by height (m) squared. A set of six skinfold thicknesses (biceps, triceps, subscapular, suprailiac, thigh and medial calf) were measured three consecutive times on the left side of the body, with a Holtain caliper (Holtain, Ltd,. Wales, UK) to the nearest 0.2 mm. The waist circumference was measured using a non-elastic tape (SECA 200; SECA, Hamburg, Germany) to the nearest 0.1 cm, according to Lohman’s anthropometric standardization reference manual [25]. In every city, the same trained investigator made all skinfold thickness measurements. For all the skinfold thickness measurements, intra-observer technical errors of measurement were smaller than 1 mm and reliability greater than 95%. Inter-observer reliability for skinfolds was higher than 90% [24]. We calculated fat mass percentage (%FM) using skinfold thickness from Slaughter’s equation [26]; which have shown to be a valid equation in adolescents [27]. FFM in kilograms was derived by subtracting fat mass from total body weight. For bioelectrical impedance analysis (BIA) measurements, a classical tetra-polar bioelectrical device was used by means of a 50 KHz BIA 101 AKERN (Akern Srl., Firenze, Italy). Standard instructions for BIA measurements were followed [28]. FFM was estimated from BIA (FFMBIA) as marker of muscle mass using validated formulas. FFMBIA (kg) for males = −9.88 + 0.65 stature^2^/resistance + 0.26 weight + 0.02 resistance; and FFMBIA (kg) for females = −11.03 + 0.70 stature^2^/resistance + 0.17 weight + 0.02 resistance [29]. Pubertal status was evaluated by experienced physicians according to the criteria of Tanner and Whitehouse [30].

### Physical activity (PA)

The Actigraph accelerometer (Actigraph MTI, model GT1M, Manufacturing, Pensacola, FL, USA) was used to measure PA. The Actigraph has been previously validated in laboratory and free-living conditions in young people [31]. Adolescents were instructed to place the monitor underneath their clothing, at the lower back, using an elastic waist band. They were asked to wear the accelerometer during the daytime for seven consecutive days, except during water-based activities. The criterion for inclusion was to record at least 8 h/d for at least 3 d (two week days and 1 weekend day). In this study, the time sampling interval (epoch) was set at 15 s. Non-wearing time was defined by bouts of at least 20 min of zero outputs. Average PA was defined as the sum of recorded counts per epoch divided by total daily registered time expressed in minutes. Time spent in light intensity PA was defined as the sum of time (in minutes) per day in which counts per minute (cpm) were 500–1,999. The time spent in moderate PA [3–6 metabolic equivalents (METs)] was calculated based upon a cut-off of 2,000-3,999 cpm. The time engaged at vigorous PA (>6 METs) was calculated based upon a cut-off of 4,000 cpm [32-35]. Also, the time spent in at least moderate intensity level (>3 METs) was calculated as the sum of time spent in moderate and vigorous PA (MVPA). The cut-offs to define the intensity categories are similar to those used in previous studies [32-36]. Moreover, this cut-offs have show to be valid in detecting recommendation of PA levels/intensities to avoid low-cardiorespiratory fitness and the excess of body fat in European adolescents [34,35].

### Family affluence scale (FAS)

The FAS is based on the concept of material conditions in the family to base the selection of items. Currie et al. [37] chose a set of items which reflected family expenditure and consumption that were relevant to family circumstances. Possessing these items was considered to reflect affluence and their lack, on the other hand, material deprivation. FAS was used in the HELENA-CSS as an index of socioeconomic status [38] which includes 4 questions answered by the adolescent: Do you have your own bedroom?; How many cars are there in your family?; How many PCs are there in your home?; Do you have internet access at home? It was defined low, medium and high socioeconomic status based on the final score obtained from the four questions. That is, a numerical value was given to each possible answer in the four questions, which increased as answers indicated higher affluence, i.e. the higher numbers of cars the higher affluence (higher value); or the higher number of PCs the higher affluence (higher value) [37,38]. Then the final score from all the questions was summed, ranging from 0 to 8. Finally, these scores were grouped in three levels: low (from 0 to 2), medium (from 3 to 5) and high (from 6 to 8).

### Statistical analysis

The data are presented as mean ± standard deviation, unless otherwise stated. To achieve normality in the residuals, waist circumference, sum of six skinfold thickness, %FM, FFM, FFMBIA and all the PA intensities were transformed to the natural logarithm.

Multiple linear regression models were used to study the associations of PA levels with both indices of fat mass and height (outcomes), after adjusting for pubertal status, FAS, country and FFM. Regression analysis was performed in two steps: Model I included pubertal status, FAS and country (entered as dummy variable) as confounders. Model II included model I plus FFM. Similarly, multiple linear regression models were used to analyze the relationships of PA levels with two markers of the muscle component (outcome) after adjusting for pubertal status, FAS, country (model I) and skinfold thickness (model II; model I plus skinfolds). The rationale for performing the model II was based on the fact that fat mass and FFM are the two main components of the body and have been classically related; thereby adjustments for each other are necessary.

The analyses were performed using the Statistical Package for Social Science (SPSS, v. 15.0 for Windows; SPSS Inc., Chicago, IL) and level of significance was set to 0.05.

## Results

Table 1 shows the descriptive characteristics of the study sample.

**Table 1:** Descriptive characteristics of European adolescents

	**All (n = 2200)**	**Males (n = 1016)**	**Females (n = 1184)**	***P *value**
Age (years)	14.7 ± 1.2	14.7 ± 1.2	14.7 ± 1.2	0.613
Puberal status (I/II/III/IV/V)^a^	1/6/22/39/32	1/8/23/37/31	0/4/21/41/34	
Weight (kg)	58.1 ± 12	60.9 ± 13.3	55.7 ± 10.2	<0.001
Height (m)	1.66 ± 0.09	1.70 ± 0.10	1.62 ± 0.07	<0.001
BMI (kg/m^2^)	21.1 ± 3.5	21.1 ± 3.6	21.2 ± 3.5	0.471
Waist circumference (mm)^b^	71.6 ± 8.4	73.6 ± 8.8	70.0 ± 7.7	<0.001
Sum of 6 skinfod thickness (mm)^b^	89.2 ±38.4	75.2 ± 37.5	101.2 ± 34.9	<0.001
% Fat mass (%)^a,b,c^	23.2 ± 9.2	19.6 ± 10.2	26.1 ± 7.1	<0.001
Fat-free mass (kg)^b,c^	44.0 ± 7.7	48.2 ± 8.4	40.6 ± 5.1	<0.001
Fat-free mass BIA (kg)^b,d^	46.0 ± 8.3	51.3 ± 8.6	41.5 ± 4.5	<0.001
Light PA (min/day)^b^	168.9 ± 41.8	173.7 ± 44.8	164.8 ± 38.5	<0.001
Moderate PA (min/day)^b^	39.5 ± 14.4	42.4 ± 15.4	37.0 ± 13.1	<0.001
Vigorous PA (min/day)^b^	18.9 ± 13.7	24.4 ± 14.7	14.2 ± 10.8	<0.001
MVPA (min/day)^b^	58.4 ± 23.8	66.7 ± 25.2	51.2 ± 19.8	<0.001
Average PA (counts/min)^b^	435.1 ± 154.6	490.0 ± 165.7	387.9 ± 126.7	<0.001

All values are mean ± standard deviation, or ^a^percentages. BMI, body mass index; PA, physical activity. Non-transformed data are presented in this table, but analyses were performed on ^b^log-transformed data.

^c^Calculated from Slaughter [26], ^d^derived from bioimpedance.

The results of the multiple linear regression models showing the association of PA levels with indices of fat mass and height after adjusting for pubertal status, FAS and country (model I) are presented in Table 2 for males and females. In males, light and moderate PA were negatively associated with height (both p < 0.001), while light PA was positively associated with skinfold thickness (p < 0.05). Vigorous PA was positively associated with height (p < 0.05). In addition, vigorous PA, MVPA and average PA were negatively associated with BMI, skinfold thickness, %FM and waist circumference (all p < 0.01). In females, light PA was negatively associated with height and positively associated with BMI (both p < 0.001). Vigorous PA and MVPA showed negative associations with BMI, skinfold thickness, %FM and waist circumference (all p < 0.01). Average PA showed negative associations with skinfold thickness, %FM and waist circumference (all p < 0.05). Further analyses including FFM as confounder (model II) did not substantially change main results, except for several intensities in which additional significances were found (model II). These additional significances were that light PA was positively associated with waist circumference (*β* = 0.066, p < 0.043 and *β* = 0.069, p < 0.006 for males and females, respectively) in addition to the previous associations. Moreover, light PA was positively associated with BMI in males (*β* = 0.108, p < 0.001), while average PA was negatively associated with BMI in females (*β* = −0.070, p < 0.007) (model II).

**Table 2:** Linear regression models of physical activity (PA) with indices of fat mass and height (Model I)

**Height**				**BMI**			**Skinfold thickness**^**a**^			**% Fat mass**^**a,b**^				**Waist circumference**^**a**^		
*Males*	*β*	*R*^*2*^	*P*	*β*	*R*^*2*^	*P*	*β*	*R*^*2*^	*P*		*β*	*R*^*2*^	*P*	*β*	*R*^*2*^	*P*
Light PA^a^	-0.200	0.423	<0.001	0.047	0.059	0.178	0.082	0.071	<0.05	0.053	0.079		0.128	0.007	0.097	0.832
Moderate PA^a^	-0.106	0.401	<0.001	-0.018	0.058	0.581	-0.040	0.067	0.229	-0.046	0.079		0.159	-0.049	0.099	0.130
Vigorous PA^a^	0.056	0.394	<0.05	-0.204	0.098	<0.001	-0.258	0.130	<0.001	-0.259	0.142		<0.001	-0.175	0.126	<0.001
MVPA^a^	-0.035	0.391	0.348	-0.112	0.069	0.001	-0.161	0.091	<0.001	-0.165	0.103		<0.001	-0.114	0.109	<0.001
Average PA^a^	-0.012	0.391	0.640	-0.095	0.066	<0.01	-0.149	0.087	<0.001	-0.155	0.100		<0.001	-0.097	0.106	<0.01
*Females*																
Light PA^a^	-0.119	0.148	<0.001	0.095	0.141	0.001	0.009	0.197	0.746	0.021	0.180		0.469	0.062	0.147	0.033
Moderate PA^a^	-0.049	0.137	0.094	0.008	0.133	0.794	-0.039	0.199	0.171	-0.036	0.180		0.205	-0.031	0.145	0.290
Vigorous PA^a^	0.052	0.147	0.074	-0.142	0.151	<0.001	-0.178	0.227	<0.001	-0.176	0.208		<0.001	-0.146	0.164	<0.001
MVPA^a^	-0.011	0.135	0.703	-0.064	0.136	<0.05	-0.115	0.209	<0.001	-0.110	0.19		<0.001	-0.092	0.151	<0.01
Average PA^a^	0.003	0.135	0.929	-0.043	0.134	0.153	-0.100	0.206	<0.001	0.095	0.187		0.001	-0.070	0.148	<0.05

*β*, standardized regression coefficients; *R*^*2*^, coefficients of determination. ^a^Log-transformed data.

Analyses were adjusted for model I (pubertal status, family affluence scale and country). BMI indicates body mass index, MVPA indicates moderate and vigorous PA.

^b^% fat mass derived from Slaughter equation [26].

Table 3 shows the association of PA levels with FFM and FFMBIA after adjusting for model I (pubertal status, FAS and country) and model II (model I plus skinfold thickness as index of fat mass). In males, light PA was negatively associated with FFM in model I and II (both p < 0.001), while vigorous PA showed positive associations with FFM in both models (both p < 0.001). Average PA was positively associated with FFM only in model II for males (p < 0.05). Moreover, light and moderate PA were negatively associated with FFMBIA in model I (p < 0.01 and p < 0.05, respectively), while only light PA maintained the association in model II (p < 0.001). Vigorous PA was positively associated with FFMBIA in model II (p < 0.05). In females, vigorous PA and average PA were positively associated with FFM in model II (p < 0.01 and p < 0.05, respectively), while only vigorous PA showed a positive association with FFMBIA in model II (p < 0.05).

**Table 3:** Linear regression models of physical activity (PA) with markers of fat-free mass (Models I and II)

**Fat free mass**^**a **^**(Model I)**				**Fat-free mass**^**a **^**(Model II)**			**FFMBIA**^**b **^**(Model I)**			**FFMBIA**^**b **^**(Model II)**		
*Males*	*β*	*R*^*2*^	*P*	*β*	*R*^*2*^	*P*	*β*	*R*^*2*^	*P*	*β*	*R*^*2*^	*P*
Light PA^c^	−0.119	0.475	<0.001	−0.124	0.477	<0.001	−0.093	0.387	0.001	−0.117	0.495	0.001
Moderate PA^c^	−0.044	0.465	0.083	−0.042	0.466	0.095	−0.059	0.383	<0.05	−0.039	0.485	0.111
Vigorous PA^c^	0.078	0.469	0.001	0.095	0.473	0.001	−0.026	0.380	0.331	0.063	0.487	<0.05
MVPA^c^	0.025	0.464	0.310	0.033	0.465	0.190	−0.041	0.381	0.124	0.016	0.484	0.521
Average PA^c^	0.046	0.465	0.062	0.054	0.467	<0.05	−0.029	0.381	0.270	0.023	0.484	0.360
*Females*												
Light PA^c^	−0.012	0.202	0.678	−0.014	0.263	0.607	0.000	0.174	0.987	−0.007	0.309	0.800
Moderate PA^c^	0.014	0.202	0.613	0.025	0.263	0.361	−0.006	0.174	0.846	0.006	0.309	0.832
Vigorous PA^c^	0.023	0.203	0.405	0.075	0.268	<0.01	−0.015	0.174	0.595	0.057	0.312	<0.05
MVPA^c^	0.019	0.202	0.507	0.051	0.265	0.065	−0.015	0.174	0.603	0.028	0.310	0.293
Average PA^c^	0.042	0.204	0.142	0.07	0.267	<0.05	0.009	0.174	0.755	0.048	0.311	0.077

Model I includes adjustment for pubertal status, family affluence scale and country.

Model II includes adjustment for model I + skinfold thickness.

*β*, standardized regression coefficients; *R*^*2*^, coefficients of determination.

BMI indicates body mass index, MVPA indicates moderate and vigorous PA.

^a^Derived from Slaughter equation [26], ^b^derived from bioimpedance [29], ^c^transformed data.

From the overall sample 456 adolescents were overweight or obese (around 20%). When the statistical analyses were repeated without including those participants, the overall results did not substantially change for most of the body composition markers, except to BMI in which the associations were weakened.

## Discussion

The main findings of our study suggest that PA is negatively associated with indices of fat mass and positively with markers of muscle component independently of each other in both genders. These associations seem to be more consistent in vigorous PA for both components of body composition.

Our results concur with recent data published from the NHANES study [11] in which the relationship between objectively measured PA and weight status (using BMI) was analyzed in children aged 6 to 17 years old (1560 girls and 1587 boys). They found that overweight and obese adolescents spent less time at moderate and vigorous PA intensities than normal weight counterparts in both genders, which is confirmed in our study where the BMI was negatively associated with PA at high intensities in males and females. On the other hand, a recent longitudinal study (5 years follow up) in 756 adolescents from Canada aged 12–13 years examined how the PA fluctuations affect several indices of fat mass (BMI, waist circumference and skinfolds) [14]. This study reported by Bélanger et al. [14] showed negative associations of PA with indices of fat mass in females, but positive associations of the same parameters in males. Our results partially concur with those found from Bélanger et al. [14] in females; however, it is of importance to highlight that none of the cited studies accounted in their analyses for markers of muscle mass such as FFM or FFMBIA, which in some cases could affect these kinds of relationships. In addition to the negative association between PA and markers of fat mass, our findings add the aspect that the associations remain significant after adjusting for several confounders including muscle components in both genders, especially in the vigorous PA intensity.

The specific role of PA on muscle components such as FFM or FFMBIA has been little studied [17]. Moliner Urdiales et al. (17) studied the association of PA by accelerometry with muscular strength and FFM in Spanish subgroup of HELENA-CSS (n = 363). They conclude that vigorous PA was positively associated with muscular strength but not with FFM assessed by Dual-energy X-ray absorptiometry after adjusting for age and pubertal status. In contrast, in the present study using the overall sample of the HELENA-CSS and adjusting for FAS, pubertal status, country and additionally to the previous study also adjusting for fat mass, vigorous PA was positively associated with FFM and FFMBIA in both genders. Differences between studies could be due to the additional adjusting for fat mass besides the inclusion of the adolescent from all the countries included in this project.

In addition, others studies have analyzed the role of specific exercise programs to maintain FFM during weigh loss interventions [18,19]. Johannsen et al. (18) analyzed the association between PA with fat mass and FFM simultaneously and observed that high levels of PA do not warrant the preservation of FFM, especially during a weight loss period. By contrast, Stiegler et al. (19) remarked the advantages of adding exercise training, and not only PA, when following a weight loss program, in order to maintain FFM and modify body composition. Unfortunately, due to the cross sectional design of this study we cannot compare our results with the findings from interventional studies. No studies simultaneously address the relationship of PA with indices of fat and muscle as well as giving gender-specific information from European adolescents.

Several complementary mechanisms potentially underlie these independent associations. PA could decrease fat mass by an increase in the total energy expenditure, while some kinds of PA, usually considered vigorous, can be more favorable to develop higher muscle mass (i.e. running or jumping). Finally, this increase in muscle mass has an additional effect on total energy expenditure due to its own metabolic requirements [18,19]. However, these mechanisms can only be hypothesized from our cross- sectional design but should be confirmed in further studies [5].

The overall findings from this research could be useful for future studies in considering the different body components and adjustments when studying the relationship of PA with body composition. Although the study is not representative enough to recommend the generalization of its results to the population, it is plausible to suggest that the interaction between PA and body components could have a similar pattern in other adolescents’ populations, which make these findings more interesting.

The present study has several limitations. Due to its cross-sectional design, the observed associations cannot be interpreted to reflect causal relationships. In addition, the fact that body composition has been measured with indirect methods (Slaughter equation for the %FM and an estimating formula from BIA for a marker of muscle mass). However, several studies considered these methods as valid and accurate tools [25,26,29] and these have been previously used for this purpose [3]. Although FFM may be considered as a marker of muscle mass, the way how it was measured could include some bias as FFM also includes bone and residual mass. An important strength is the large and heterogeneous sample with gender-specific information, to control for several confounders including indices of fat mass and muscle and the use of accelerometers to measure PA.

## Conclusion

The present study suggests that PA, especially vigorous PA, has an important influence on indices of fat mass and the muscular component independent of confounders. Future studies should focus not only on the classical relationship between PA and fat mass, but also on PA and muscular components, analyzing the independent role of both with the different PA intensities. Further intervention studies to analyze the effects of different PA-exercise programs on fat and muscle components at the same time are needed.

## Appendix

### HELENA Study Group

Co-ordinator: Luis A. Moreno.

Core Group members: Luis A. Moreno, Fréderic Gottrand, Stefaan De Henauw, Marcela González-Gross, Chantal Gilbert.

Steering Committee: Anthony Kafatos (President), Luis A. Moreno, Christian Libersa, Stefaan De Henauw, Jackie Sánchez, Fréderic Gottrand, Mathilde Kersting, Michael Sjöstrom, Dénes Molnár, Marcela González-Gross, Jean Dallongeville, Chantal Gilbert, Gunnar Hall, Lea Maes, Luca Scalfi.

Project Manager: Pilar Meléndez.

1. Universidad de Zaragoza (Spain). Luis A. Moreno, Jesús Fleta, José A. Casajús, Gerardo Rodríguez, Concepción Tomás, María I. Mesana, Germán Vicente-Rodríguez, Adoración Villarroya, Carlos M. Gil, Ignacio Ara, Juan Revenga, Carmen Lachen, Juan Fernández Alvira, Gloria Bueno, Aurora Lázaro, Olga Bueno, Juan F. León, Jesús Mª Garagorri, Manuel Bueno, Juan Pablo Rey López, Iris Iglesia, Paula Velasco, Silvia Bel-Serrat, Luis Gracia-Marco, Theodora Mouratidou, David Jiménez-Pavón.

2. Consejo Superior de Investigaciones Científicas (Spain). Ascensión Marcos, Julia Wärnberg, Esther Nova, Sonia Gómez, Esperanza Ligia Díaz, Javier Romeo, Ana Veses, Mari Angeles Puertollano, Belén Zapatera, Tamara Pozo.

3. Université de Lille 2 (France). Laurent Beghin, Christian Libersa, Frédéric Gottrand, Catalina Iliescu, Juliana Von Berlepsch.

4. Research Institute of Child Nutrition Dortmund, Rheinische Friedrich-Wilhelms-Universität Bonn (Germany) Mathilde Kersting, Wolfgang Sichert-Hellert, Ellen Koeppen.

5. Pécsi Tudományegyetem (University of Pécs) (Hungary). Dénes Molnar, Eva Erhardt, Katalin Csernus, Katalin Török, Szilvia Bokor, Mrs. Angster, Enikö Nagy, Orsolya Kovács, Judit Répasi.

6. University of Crete School of Medicine (Greece).Anthony Kafatos, Caroline Codrington, María Plada, Angeliki Papadaki, Katerina Sarri, Anna Viskadourou, Christos Hatzis, Michael Kiriakakis, George Tsibinos, Constantine Vardavas Manolis Sbokos, Eva Protoyeraki, Maria Fasoulaki.

7. Institut für Ernährungs- und Lebensmittelwissenschaften – Ernährungphysiologie. Rheinische Friedrich Wilhelms Universität (Germany).Peter Stehle, Klaus Pietrzik, Marcela González-Gross, Christina Breidenassel, Andre Spinneker, Jasmin Al-Tahan, Miriam Segoviano, Anke Berchtold, Christine Bierschbach, Erika Blatzheim, Adelheid Schuch, Petra Pickert.

8. University of Granada (Spain). Manuel J. Castillo Garzón, Ángel Gutiérrez Sáinz, Francisco B. Ortega Porcel, Jonatan Ruiz Ruiz, Enrique García Artero, Vanesa España Romero, David Jiménez Pavón, Cristóbal Sánchez Muñoz, Victor Soto, Palma Chillón, Jose M. Heredia, Virginia Aparicio, Pedro Baena, Claudia M. Cardia, Ana Carbonell.

9. Istituto Nazionalen di Ricerca per gli Alimenti e la Nutrizione (Italy). Davide Arcella, Giovina Catasta, Laura Censi, Donatella Ciarapica, Marika Ferrari, Cinzia Le Donne, Catherine Leclerq, Luciana Magrì, Giuseppe Maiani, Rafaela Piccinelli, Angela Polito, Raffaela Spada, Elisabetta Toti.

10. University of Napoli "Federico II" Dept of Food Science (Italy) Luca Scalfi, Paola Vitaglione, Concetta Montagnese.

11. Ghent University (Belgium). Ilse De Bourdeaudhuij, Stefaan De Henauw, Tineke De Vriendt, Lea Maes, Christophe Matthys, Carine Vereecken, Mieke de Maeyer, Charlene Ottevaere, Inge Huybrechts.

12. Medical University of Vienna (Austria). Kurt Widhalm, Katharina Phillipp, Sabine Dietrich, Birgit Kubelka Marion Boriss-Riedl.

13. Harokopio University (Greece). Yannis Manios, Eva Grammatikaki, Zoi Bouloubasi, Tina Louisa Cook, Sofia Eleutheriou, Orsalia Consta, George Moschonis, Ioanna Katsaroli, George Kraniou, Stalo Papoutsou, Despoina Keke, Ioanna Petraki, Elena Bellou, Sofia Tanagra, Kostalenia Kallianoti, Dionysia Argyropoulou, Katerina Kondaki, Stamatoula Tsikrika, Christos Karaiskos.

14. Institut Pasteur de Lille (France). Jean Dallongeville, Aline Meirhaeghe.

15. Karolinska Institutet (Sweden). Michael Sjöstrom, Patrick Bergman, María Hagströmer, Lena Hallström, Mårten Hallberg, Eric Poortvliet, Julia Wärnberg, Nico Rizzo, Linda Beckman, Anita Hurtig Wennlöf, Emma Patterson, Lydia Kwak, Lars Cernerud, Per Tillgren, Stefaan Sörensen.

16. Asociación de Investigación de la Industria Agroalimentaria (Spain). Jackie Sánchez-Molero, Elena Picó, Maite Navarro, Blanca Viadel, José Enrique Carreres, Gema Merino, Rosa Sanjuán, María Lorente, María José Sánchez, Sara Castelló.

17. Campden BRI (United Kingdom). Chantal Gilbert, Sarah Thomas, Elaine Allchurch, Peter Burguess.

18. SIK - Institutet foer Livsmedel och Bioteknik (Sweden). Gunnar Hall, Annika Astrom, Anna Sverkén, Agneta Broberg.

19. Meurice Recherche & Development asbl (Belgium).Annick Masson, Claire Lehoux, Pascal Brabant, Philippe Pate, Laurence Fontaine.

20. Campden & Chorleywood Food Development Institute (Hungary). Andras Sebok, Tunde Kuti, Adrienn Hegyi.

21. Productos Aditivos SA (Spain). Cristina Maldonado, Ana Llorente.

22. Cárnicas Serrano SL (Spain). Emilio García.

23. Cederroth International AB (Sweden). Holger von Fircks, Marianne Lilja Hallberg, Maria Messerer

24. Lantmännen Food R&D (Sweden). Mats Larsson, Helena Fredriksson, Viola Adamsson, Ingmar Börjesson.

25. European Food Information Council (Belgium).Laura Fernández, Laura Smillie, Josephine Wills.

26. Universidad Politécnica de Madrid (Spain). Marcela González-Gross, Jara Valtueña, Ulrike Albers, Raquel Pedrero, Agustín Meléndez, Pedro J. Benito, David Cañada, David Jiménez-Pavón, Alejandro Urzanqui, Juan Carlos Ortiz, Francisco Fuentes, Juan José Gómez Lorente, Rosa María Torres, Paloma Navarro.

## Abbreviations

BIA: Bioelectrical impedance analysis; BMI: Body mass index; FAS: Family affluence scale; FFM: Fat-free mass; FMI: Fat mass index; %FM: Fat mass percentage; FFMBIA: FFM estimated from BIA; HELENA-CSS: Healthy lifestyle in Europe by nutrition in adolescence cross-sectional study; MVPA: Moderate and vigorous PA; PA: Physical activity; SPSS: Statistical package for social science.

## Competing interests

The authors declare that they have no competing interests.

## Authors’ contributions

DJP, AFV, and LAM contributed to the concept and design of the study. DJP, AFV, UA, RP, MCG, AP, JV, YM, AK, DM, MS and LAM contributed to the conduct of the study. DJP, AFV, UA, MCG and LAM contributed to the analysis and interpretation of data. DJP, AFV, RP, AP, JV, YM, AK, DM, MS and LAM contributed to drafting the manuscript. All authors critically reviewed the manuscript, read the manuscript and have had important critical input before the final approval. DJP is the guarantor.

## Pre-publication history

The pre-publication history for this paper can be accessed here:

http://www.biomedcentral.com/1471-2458/13/667/prepub
